# Vegetation plot and trait data from phonolitic and basaltic rocks on La Palma (Canary Islands, Spain)

**DOI:** 10.1016/j.dib.2021.107229

**Published:** 2021-06-15

**Authors:** Anna Walentowitz, David Kienle, Leyla Sungur, Carl Beierkuhnlein

**Affiliations:** a Biogeography, University of Bayreuth, Bayreuth 95440, Germany; b Bayreuth Center of Ecology and Environmental Research BayCEER, University of Bayreuth, Bayreuth 95440, Germany; c Geographical Institute Bayreuth, GIB, University of Bayreuth, Bayreuth 95440, Germany

**Keywords:** Biodiversity, *Cheirolophus junonianus*, Endemic species, Habitat islands, Oceanic islands, Phonolite, Species richness

## Abstract

Geodiversity promotes biodiversity by increasing habitat heterogeneity. In times of a global biodiversity decline, data about diversity on such geological elements gains importance, also regarding conservation and restoration. In the Canary Islands, phonolitic rocks are geological elements of volcanic origin that represent additional habitat for species. In the southern part of the island La Palma, phonolite rocks can be encountered in matrices of young lava. We provide biotic and abiotic records from 60 plots of 2 m × 2 m, sampled on phonolitic and neighbouring basaltic outcrops at four different sites that cover a wide environmental gradient. The recorded parameters were species abundances (percentage cover), plant individuals' frequency (number of plant individuals per plot and species), plant growth height (in cm), and canopy diameter (in cm). Additionally, lichen cover (percentage cover) was estimated. To describe site specific characteristics, we recorded plot surface dynamics (3D rock surface), aspect, and inclination. This data set covers vegetation and trait data comparing phonolites and basalts on La Palma. It can be used for island biogeography, vegetation ecology and conservation sciences to help support fundamental research related to insular biodiversity and endemism, and to identify priority areas for protection and preservation in the Canary Islands.

## Specifications Table

**Table:** 

Subject	Biodiversity
Specific subject area	The vegetation plot data provided is of interest for island biogeography, vegetation ecology and relevant for questions related to insular endemism and biodiversity.
Type of data	Table
How data were acquired	Field observations and measurements.
Data format	Raw
Parameters for data collection	We collected data from 60 square 2 m × 2 m vegetation plots sampled on phonolite and on basaltic rock. In each plot, species composition, abundance (estimated percentage cover), frequency (number of individuals per species), plant height (cm), and canopy diameter (cm) were sampled. Additional information about lichens cover was measured. Finally, essential geomorphological information were collected for each plot: surface rugosity (3D rock surface), aspect and inclination.
Description of data collection	Data collection took place in spring 2018 (10–15. March). The four phonolites were selected based on Middlemost (1972) [1] and adjacent basaltic outcrops of comparable size were chosen. Biotic parameters: • Plant species were identified in the field or, in unclear cases, at our field station. Taxonomy follows the standards of “Plants of the World Online” (POWO 2019) [2]. Additional species’ information, namely family, if perennial/annual and status (single- or multi-island endemic, native, introduced) were collected from Muer et al. (2016) [3] and the ATLANTIS database [4]. • Absolute frequency was calculated for each species sampled in each 2 m × 2 m plot. • Plant cover per species and total lichen cover were estimated as a fraction of the plot area and expressed as percentages. • Plant height for each individual was measured from the stem base to the top of the plant (in cm). Canopy diameter was measured as the maximum lateral spread of a plant (in cm). Abiotic parameters: • Plot surface rugosity (3D rock surface) was measured by placing a thread diagonally across a plot as close to the rock surface as possible. Both diagonals were measured, from the top-left to the bottom-right corner and from the top-right to the bottom-left corner. The longer the thread length needed to cover the diagonals, the higher the 3D rock surface dynamics. • Aspect was recoded with a compass and expressed in degree. • Inclination was estimated and expressed in degree.
Data source location	Region: Southern La Palma (Canary Islands) Country: Spain Coordinates of sampling sites: 28°35′01.1″N 17°52′10.2″W 28°30′56.4″N 17°50′00.1″W 28°28′51.6″N 17°51′23.5″W 28°28′40.4″N 17°51′31.8″W
Data accessibility	With the article

## Value of the Data

• Habitat island studies can benefit from phonolite rocks as these island-like systems are volcanic outcrops within a matrix of surrounding basaltic rocks.
• Island biogeographers can benefit from the dataset to answer questions related to insular biodiversity and endemism, and conservationists to identify priority areas for protection and preservation.
• Global extinction risk studies are mostly focused on endemic species with small range sizes, such as the isolated habitats and populations in this study.
• Research related to the role of geodiversity for biodiversity, especially on oceanic islands, might benefit from our dataset.

## 1 Data Description

**Fig. 1:**
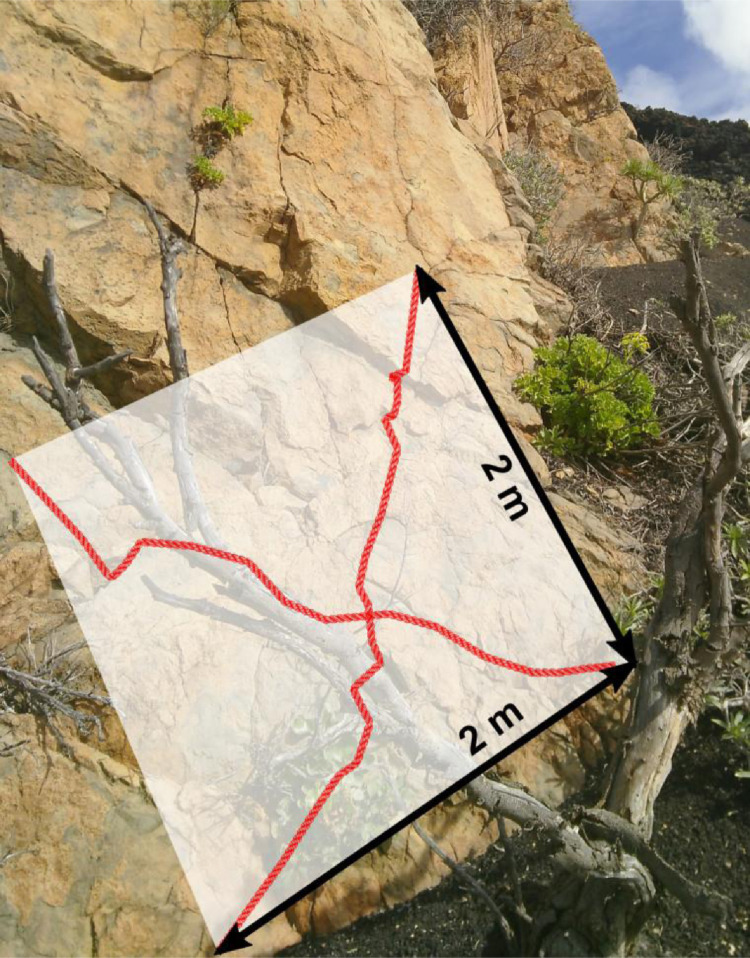
Illustration of our plot design. The two black arrows indicate the plot area (2 × 2 m) in relation to the rock's inclination. The two red lines illustrate how we estimated the rock's rugosity (3D surface) using a raffia thread following the rocks surface from the bottom-left to the top-right and from the top-left to the bottom-right corner.

We provide raw data in the form of a data table. Details about the investigated parameters are available in Table 1. An illustration of the sampling design can be found in Fig. 1.

**Table 1:** Table describing the investigated parameters available as raw data. The column *Plot* indicates a running plot ID. *Longitude* and *Latitude* are UTM coordinates (zone 28N). *Rugosity1* and *Rugosity2* are the two measured values of rock rugosity (3D surface). *Lichen_cover* is the estimated total cover of lichen for the whole plot. *Species* are species names of the individual sampled individuals. *Species_cover* indicates the estimated value of the cover of all individuals of the same species on the plot. *Height* is the measured plant height of all individuals in cm. *Canopy_diameter* is the measured canopy diameter of all individuals in cm. *Rock_type* indicates whether the plot is on a phonolitic or basaltic rock. “B” stands for basaltic and "P” for phonolitic rock. *Perennial* indicates if a species is perennial (“1”) or not (“0”). Species status indicates if a species is endemic to the Canary Islands (“MIE”, multi-island endemic) to La Palma only (“SIE”, single-island endemic), native (“nat.”), or introduced ("intr.”).

Column name	Data type	Unit	Range	Measurement method	Reference of the method	Levels of factors
Plot	Nominal-scaled	–	–	Defined	-	-
Longitude	Ratio-scaled	m	218761–222706	GPS	EPSG 32628	-
Latitude	Ratio-scaled	m	3158040–3165299	GPS	EPSG 32628	-
Aspect	Interval-scaled (circular data)	°	0–355	Compass with inclinometer	-	-
Inclination	Interval-scaled (circular data)	°	0–90	Compass with inclino-meter	-	-
Rugosity1	Ratio-scaled	cm	245–735	Raffia thread & measuring tape	Fig. 1	-
Rugosity2	Ratio-scaled	cm	258–565	Raffia thread & measuring tape	Fig. 1	-
Lichens_ cover	Ratio-scaled	%	1–90	Estimation	-	-
Species	Nominal-scaled	-	–	Own knowledge and literature work	[2,3]	-
Species_ cover	Ratio-scaled	%	1–30	Estimation	-	-
Height	Ratio-scaled	cm	0–550	Measuring tape	-	-
Canopy_ diameter	Ratio-scaled	cm	0.1–750	Measuring tape	-	-
Rock_type	Binary-scaled	–	–	Knowledge	-	“B”, “P”
Perennial	Binary-scaled	–	–	Knowledge	-	“1”, “0”
Species status	Nominal-scaled	–	–	Literature work	[3]	“SIE”, “MIE”, “intr.”, “nat.”

## 2 Experimental Design, Materials and Methods

### 2.1 Study design

**Fig. 2:**
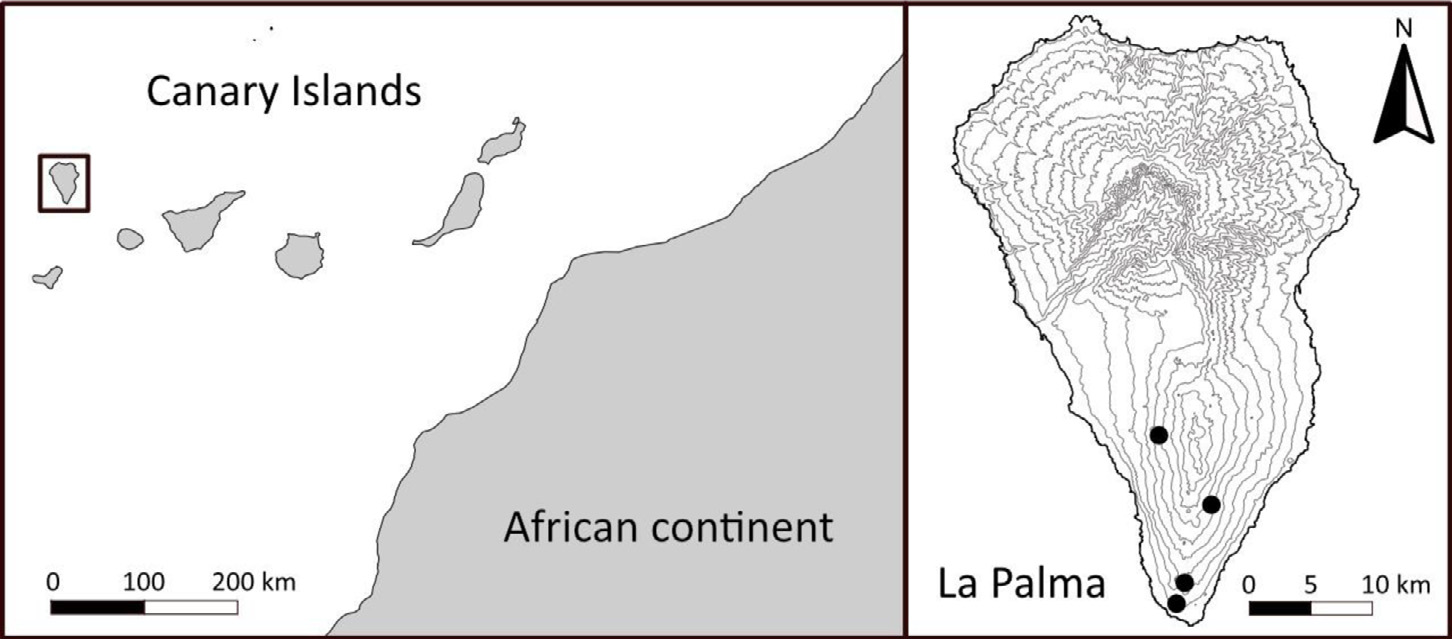
Data collection took place in the Canary Islands (left) on the island of La Palma (right) at four sites in the southern part of the island (black dots).

The study focuses on phonolitic rocks and adjacent basaltic outcrops on southern La Palma. We selected four sampling sites based on geologic information about locations of phonolites given by Middlemost [1] that cover a wide environmental gradient on the island (Fig. 2). At all sites, phonolitic and adjacent basaltic outcrops were sampled. We placed plots on neighbouring rocks of comparable size. At all sites and on both phonolitic and basalitc rocks we measured a set of vegetational and environmental parameters to account for plot characteristics.

### 2.2 Data collection

The data collection took place in spring 2018 (10–15. March) and have previously been used for one publication [5]. We collected data from 60 square 2 m × 2 m vegetation plots sampled on phonolite and 60 square 2 m × 2 m vegetation plots sampled on basalt, respectively. (Total of 120 plots with 15 plots per outcrop at four locations on phonolite and four respective locations on basalt) The plots were randomly selected for each outcrop. The placement of plots was restricted to the range within accessibility. All plots were marked with GPS coordinates. The species (vascular plants) were identified and all individuals per species (frequency) were counted within each plot. Plant height and canopy diameter for all individuals were measured with a measuring tape (in cm). The total cover of each species and the total cover for existing lichens were estimated.

We collected basic topographic information for each plot. Aspect was determined with the help of a geologic compass (expressed in degree). Northness and eastness were calculated as the cosine and sinus from the aspect measurements. Inclination was estimated and raffia threats were chosen to measure the rugosity (3D rock surface as a proxy for plot surface dynamics). These were laid out as close as possible to the surface, arranged from the top-left to the bottom-right and from the bottom-left to the top-right corner of each plot (Fig. 1).

## Ethics Statement

We had a permit by the Cabildo de La Palma to take samples on the Roque de Teneguía, an archaeological site. We determined the plant species and took the measurements on this rock of high cultural importance of the Canary history with special consideration and care.

## CRediT Author Statement

**David Kienle, Anna Walentowitz** and **Leyla Sungur:** Conceptualization, Methodology, Data curation and preparation, Writing and editing; **Carl Beierkuhnlein:** Conceptualization, Methodology, Supervision and editing.

## Declaration of Competing Interest

The authors declare that they have no known competing financial interests or personal relationships which have or could be perceived to have influenced the work reported in this article.

## Appendix Supplementary materials

Image, application 1
